# Platinum single-atom and cluster catalysis of the hydrogen evolution reaction

**DOI:** 10.1038/ncomms13638

**Published:** 2016-11-30

**Authors:** Niancai Cheng, Samantha Stambula, Da Wang, Mohammad Norouzi Banis, Jian Liu, Adam Riese, Biwei Xiao, Ruying Li, Tsun-Kong Sham, Li-Min Liu, Gianluigi A. Botton, Xueliang Sun

**Affiliations:** 1 Department of Mechanical and Materials Engineering, University of Western Ontario, London, Ontario, Canada N6A 5B9; 2 Department of Materials Science and Engineering, McMaster University, Hamilton, Ontario, Canada L8S 4L8; 3 Beijing Computational Science Research Center, Beijing 100193, China; 4 Department of Chemistry, University of Western Ontario, London, Ontario, Canada N6A 5B7; 5 Brockhouse Institute for Materials Research, McMaster University, Hamilton, Ontario, Canada L9S 4M1; 6 Canadian Centre for Electron Microscopy, McMaster University, Hamilton, Ontario, Canada L8S 4M1

## Abstract

Platinum-based catalysts have been considered the most effective electrocatalysts for the hydrogen evolution reaction in water splitting. However, platinum utilization in these electrocatalysts is extremely low, as the active sites are only located on the surface of the catalyst particles. Downsizing catalyst nanoparticles to single atoms is highly desirable to maximize their efficiency by utilizing nearly all platinum atoms. Here we report on a practical synthesis method to produce isolated single platinum atoms and clusters using the atomic layer deposition technique. The single platinum atom catalysts are investigated for the hydrogen evolution reaction, where they exhibit significantly enhanced catalytic activity (up to 37 times) and high stability in comparison with the state-of-the-art commercial platinum/carbon catalysts. The X-ray absorption fine structure and density functional theory analyses indicate that the partially unoccupied density of states of the platinum atoms' 5*d* orbitals on the nitrogen-doped graphene are responsible for the excellent performance.

## Abstract (web summary)

Downsizing platinum based nanocatalysts has the twin advantages of lower platinum usage and increased activity per platinum atom. Here, the authors report an atomic layer deposition technique for single platinum atom catalyst fabrication and assess their hydrogen evolution activity.

## Graphical abstract

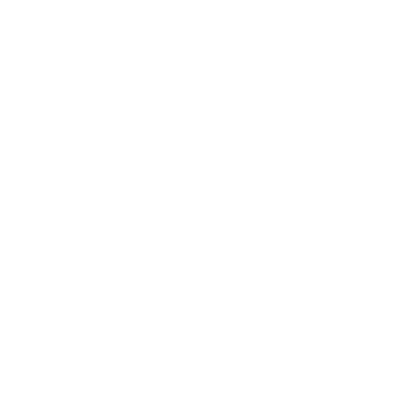

Securing renewable and reliable sources of clean energy is one of the world's foremost challenges. Addressing this challenge is not only critical for the global economy but will also aid in the mitigation of environmental and health hazards caused by fossil fuels^1^. Hydrogen is the cleanest fuel available and is believed to be one of the most promising energy sources of the twenty-first century^2^,^3^. However, the majority of the hydrogen produced today is derived from steam-reformed methane, which is sourced from fossil reserves and produces a substantial amount of CO_2_ (ref. 4). The production of hydrogen from water electrolysis is a promising alternative to the current CO_2_-emitting fossil fuel-based energy systems^5^,^6^.

Platinum (Pt)-based catalysts are generally considered to be the most effective electrocatalysts for the hydrogen evolution reaction (HER)^5^,^7^. Unfortunately, Pt is expensive and scarce, limiting the commercial potential for such catalysts. The development of active, stable and inexpensive electrocatalysts for water splitting is a key step in the realization of a hydrogen economy, which is based on the use of molecular hydrogen for energy storage. Significant effort has been devoted to the search of non-precious-metal-based HER catalysts, including sulfide-based materials^8^,^9^,^10^,^11^, and C_3_N_4_ (refs 12, 13, 14). Although these candidate materials show promising activities for the HER, the activities of these catalysts in their present form are insufficient for industrial applications^15^.

To overcome the challenges associated with the Pt HER catalysts and to drive the cost of H_2_ production from water electrolysis down, it is very important to markedly decrease the Pt loading and increase the Pt utilization efficiency. Currently, supported Pt nanoparticles (NPs) are typically used to promote Pt activity towards the HER. Unfortunately, the geometry of the NPs limit the majority of the Pt atoms to the particle core, deeming them ineffective, as only surface atoms are involved in the electrochemical reaction^16^. Reducing the size of the Pt NPs to clusters or even single atoms could significantly decrease the noble metal usage and increase their catalytic activity, which is highly desirable to enhance the Pt utilization and decrease the cost of the electrocatalysts^17^. It has been shown that single Pt atoms dispersed on an FeO_*x*_ surface have a higher catalytic activity for CO oxidation compared with the corresponding Pt NPs^18^. Moreover, the single-atom catalysts also exhibited a significantly improved catalytic activity towards methanol oxidation, up to 10 times greater than the state-of-the-art commercial carbon-supported Pt (Pt/C) catalysts^19^.

Controlled and large-scale synthesis of stable single atoms and clusters remains a considerable challenge due to the natural tendency for metal atoms to diffuse and agglomerate, resulting in the formation of larger particles^20^,^21^. In practical applications, it is required that the single atoms not only have a high activity but also exhibit a satisfactory stability^17^,^22^,^23^. Moreover, it is also desired to produce a high density of single atoms to meet the practical applications. Consequently, an ideal single-atom catalyst must have a high activity, a high stability and a high density. Thus, we need to discover an effective means to synthesize this ideal single-atom catalyst. In this paper, the atomic layer deposition (ALD) technique was utilized, as it has been proven to be a powerful tool for large-scale synthesis of stable single-atom and cluster catalysts^19^,^24^. ALD has the ability to precisely control the size and distribution of particles on a substrate by using sequential and self-limiting surface reactions^25^,^26^,^27^.

In this work, we fabricate single platinum atoms and clusters supported on nitrogen-doped graphene nanosheets (NGNs) for the HER using the ALD technique, resulting in the utilization of nearly all the Pt atoms. The size and density of the Pt catalysts on the NGNs are precisely controlled by simply adjusting the number of ALD cycles. The Pt atoms and clusters on the NGNs show much greater activity for the HER in comparison with conventional Pt NP catalysts.

## Results

### Electron microscopy characterization

**Figure 1:**
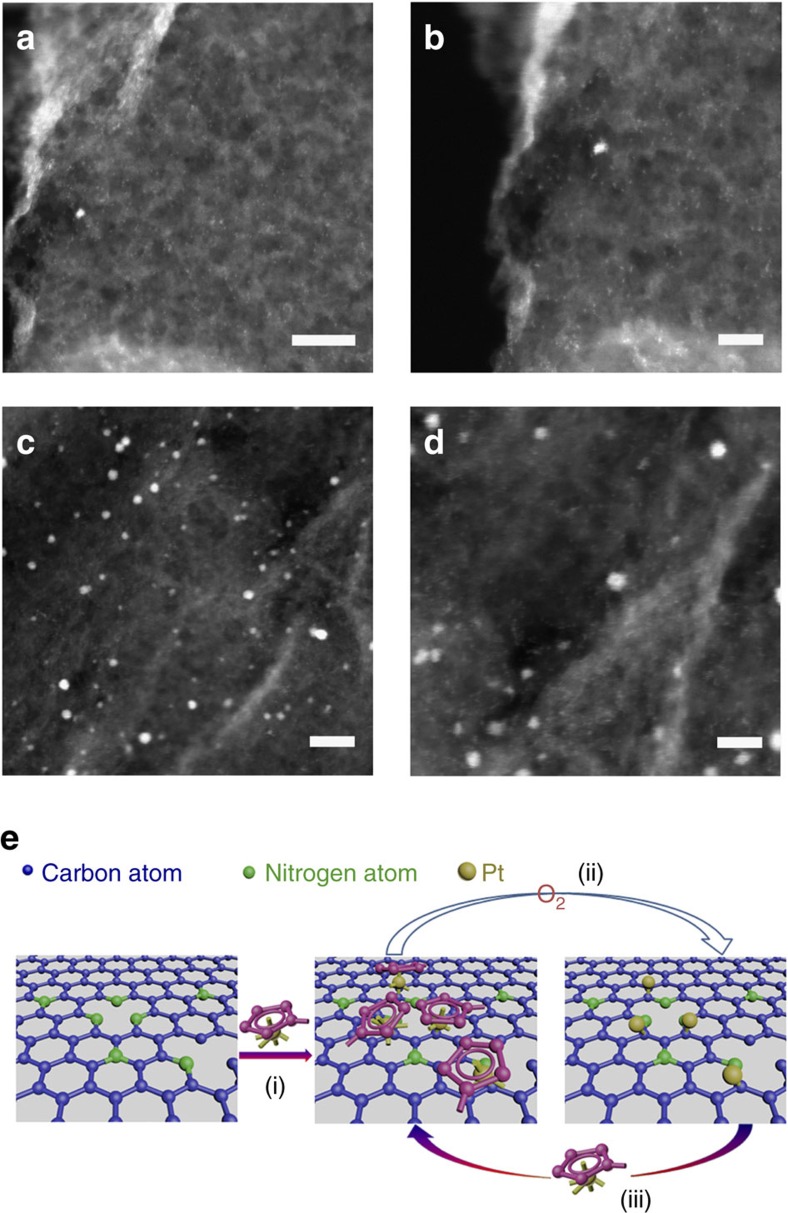
ADF STEM images and schematic illustration of the Pt ALD mechanism on NGNs. ADF STEM images of ALDPt/NGNs samples with (**a**,**b**) 50 and (**c**,**d**) 100 ALD cycles. Scale bars, 10 nm (**a**,**c**); 5 nm (**b**,**d**). (**e**) Schematic illustration of the Pt ALD mechanism on NGNs. The ALD process includes the following: the Pt precursor (MeCpPtMe_3_) first reacts with the N-dopant sites in the NGNs (i). During the following O_2_ exposure, the Pt precursor on the NGNs is completely oxidized to CO_2_ and H_2_O, creating a Pt containing monolayer (ii). These two processes (i and ii) form a complete ALD cycle. During process (ii), a new layer of adsorbed oxygen forms on the platinum surface, which provides functional groups for the next ALD cycle process (iii).

The morphology of the ALD Pt on NGNs with 50 and 100 cycles (denoted hereafter as ALD50Pt/NGNs and ALD100Pt/NGNs, respectively) were characterized by annular dark field (ADF) imaging with aberration-corrected scanning transmission electron microscopy (STEM). The high spatial resolution allowed for the precise determination of the size and distribution of the individual metal atoms, thus providing local structural information about the metal species on the NGN supports^24^,^28^,^29^. It can be clearly observed in Fig. 1a,b that the numerous individual Pt atoms (bright spots), as well as very small Pt clusters are uniformly dispersed on the NGNs' surface for the ALD50Pt/NGNs catalysts. Through examination of multiple NGNs, small NPs were also detected on this sample (Supplementary Fig. 1a). After 100 ALD cycles many single Pt atoms and small clusters were still present, yet it appears as though some Pt clusters have grown to form NPs at a larger quantity than observed for the 50 ALD cycles (Fig. 1c,d and Supplementary Fig. 1b). These findings suggest that ALD is a suitable technique for preparing single atoms/clusters on nitrogen-doped graphene supports. As shown in Fig. 1e, during the ALD process, the Pt precursor (MeCpPtMe_3_) first reacts with the NGNs as influenced by the N-dopant. The chemical bonding between the Pt precursor and NGNs ensures a strong interaction between the deposited material and the support^30^. The Pt loading after ALD was confirmed using inductively coupled plasma-atomic emission spectroscopy, in which a loading of 2.1 and 7.6 wt% was obtained for the 50 and 100 ALD cycled samples, respectively.

### Catalytic performance and stability

**Figure 2:**
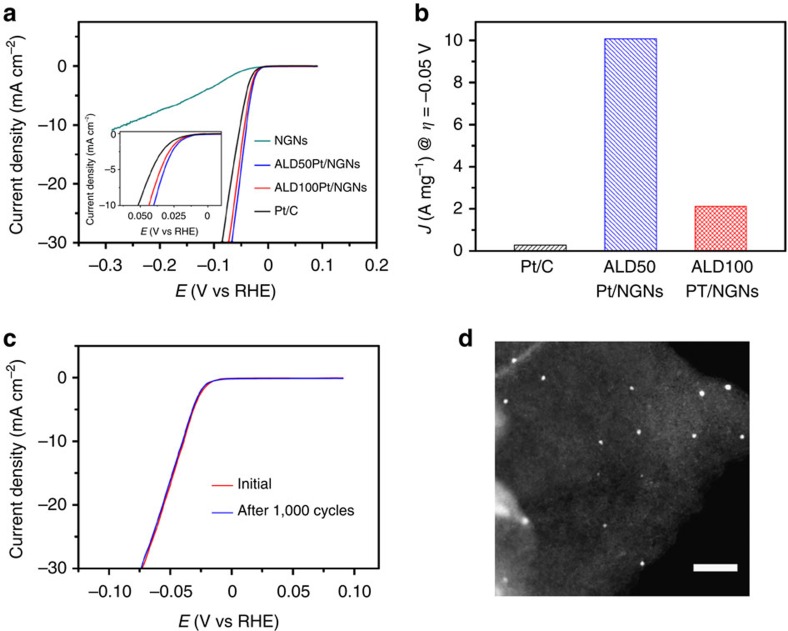
Electrocatalytic properties. (**a**) The HER polarization curves for ALDPt/NGNs and Pt/C catalysts were acquired by linear sweep voltammetry with a scan rate of 2 mV s^−1^ in 0.5 M H_2_SO_4_ at room temperature. N_2_ was purged before the measurements. The inset shows the enlarged curves at the onset potential region of the HER for the different catalysts. (**b**) Mass activity at 0.05 V (versus RHE) of the ALDPt/NGNs and the Pt/C catalysts for the HER. (**c**) Durability measurement of the ALD50Pt/NGNs. The polarization curves were recorded initially and after 1,000 cyclic voltammetry sweeps between +0.4 and −0.15 V (versus RHE) at 100 mV s^−1^ in 0.5 M H_2_SO_4_ at a scan rate of 2 mV s^−1^. (**d**) ADF STEM images of ALD50Pt/NGNs samples after ADT; scale bar, 20 nm.

The HER activity of the ALDPt/NGNs with 50 and 100 cycles were measured in comparison with commercial Pt/C catalysts by conducting linear sweep voltammetry measurements in 0.5 M H_2_SO_4_ at room temperature. The NGNs without Pt catalysts exhibited a poor HER activity (Fig. 2a), which is consistent with previous studies^12^. As shown in Fig. 2a, both ALDPt/NGN and Pt/C catalysts exhibited excellent catalytic activities towards the HER, with negligible overpotentials. Importantly, the ALDPt/NGN catalysts exhibited much higher HER activities than that of the commercial Pt/C catalysts (Fig. 2a). It was observed that the HER catalytic activity of the ALDPt/NGNs decreased with an increased number of ALD cycles. This is likely attributed to the increased formation of clusters or NPs in the sample with 100 ALD cycles. Supplementary Fig. 2 showed consistent results with previous studies, as the Pt/C catalyst achieved a Tafel slope of 31 mV dec^−1^ (ref. 31). Following the same measurement parameters, a smaller Tafel slope than the Pt/C catalysts was achieved for the ALDPt/NGNs catalysts at 29 mV dec^−1^. The specific activity for each catalyst was calculated from the polarization curves by normalizing the current with the geometric area of the electrode. As shown in Supplementary Fig. 3, the HER activities for the ALD50Pt/NGNs, ALD100Pt/NGNs and Pt/C catalysts are 16, 12.9 and 8.2 mA cm^−2^, respectively, at the overpotential of 0.05 V. Furthermore, normalized to the Pt loading (Fig. 2b), the mass activity of the HER for the ALD50Pt/NGNs catalysts at the overpotential of 0.05 V was 10.1 A mg^−1^. Remarkably, the mass activity of the ALD50Pt/NGNs catalyst was 7.8 times greater than the ALD100Pt/NGNs catalyst (2.12 A mg^−1^) and 37.4 times greater than the Pt/C catalyst (0.27 A mg^−1^). These findings suggest that the single Pt atoms and clusters can significantly increase the Pt utilization activity in comparison to their NP counterparts^18^,^32^, with the additional benefit of decreasing the cost of the catalyst for the HER.

The long-term stability of the ALDPt/NGNs and Pt/C catalysts were examined by extended electrolysis at fixed potentials. Supplementary Fig. 4 shows that the ALDPt/NGNs catalysts appear stable at 0.04 V versus reversible hydrogen electrode (RHE), while the current density of the Pt/C catalysts degraded with time at the same operating conditions. The lower stability of the Pt/C catalysts may be due to a weak interaction between the supported Pt particles and the C substrate^33^, resulting in the detachment and/or agglomeration of the Pt NPs. Accelerated degradation tests (ADTs) were also adopted to evaluate the durability of the Pt atom/cluster catalysts for the HER activity. As exhibited in Fig. 2c, the ALD50Pt/NGNs catalysts' polarization curve after 1,000 cycles retained a similar performance to the initial test, resulting in a loss of only 4% of its initial current density at an overpotential of 0.05 V (Supplementary Fig. 5). The significant stability of the ALD50Pt/NGNs catalysts can be observed when comparing its performance with the ALD100Pt/NGNs catalysts (∼10% loss) and the Pt/C catalysts (19% loss) under the same measurement conditions (Supplementary Figs 5 and 6).

STEM images acquired from the ALDPt/NGNs after the ADTs (Fig. 2d and Supplementary Fig. 7) showed that there was only a slight increase in the Pt size with no obvious aggregation. This further supports the excellent stability observed in the activity from the ALDPt/NGNs catalysts during the ADTs for the HER. In contrast, according to high-resolution transmission electron microscope images, the Pt/C catalysts (Supplementary Fig. 8) coalesced into larger particles (marked with white circles in Supplementary Fig. 8b) with the average size of Pt NPs increasing from 4.2 to 5.5 nm after the ADT. The growth of the NPs may be caused by Pt migration due to weak interactions between the catalyst and the support^34^,^35^. As shown in Supplementary Fig. 9, the negative potentials of a HER cathode are generally not conducive for electro-oxidation of the support material. In addition, Pt is one of the most stable electrocatalyst materials^36^,^37^, and Pt oxidation/dissolution is typically not observed at potentials <0.85 V versus RHE in acidic conditions, thus the Pt and the C supports are stable under HER conditions. This suggests that the higher stability of the Pt atoms/clusters observed on the ALDPt/NGNs samples could be ascribed to the stronger interactions between the Pt and the NGNs supports compared to the Pt/C catalysts.

### X-ray absorption spectroscopy studies

**Figure 3:**
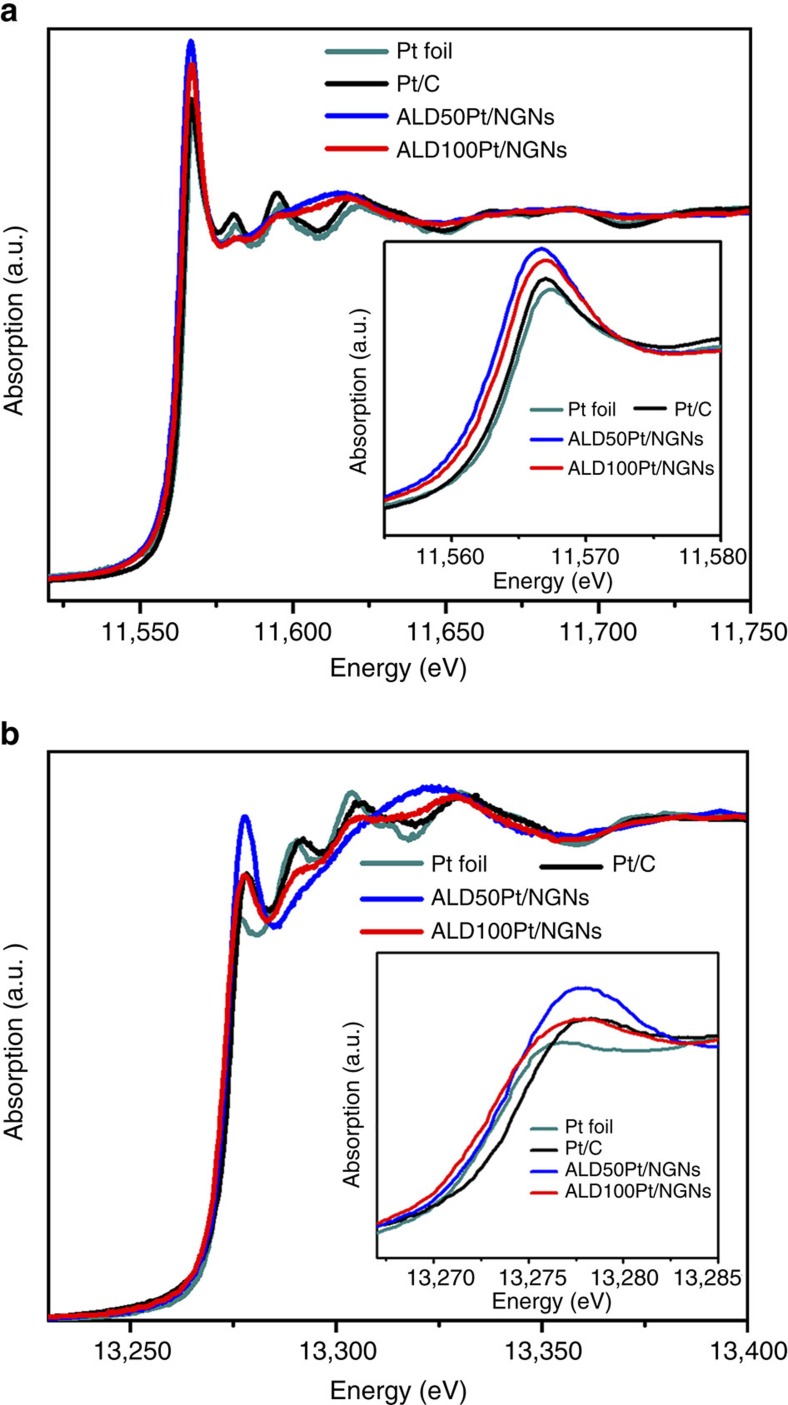
X-ray absorption studies. (**a**) The normalized XANES spectra at the Pt L_3_-edge of the ALDPt/NGNs, Pt/C catalysts and Pt foil. The inset shows the enlarged spectra at the Pt L_3_-edge. (**b**) The normalized XANES spectra at the Pt L_2_-edge of ALDPt/NGNs, Pt/C catalysts and Pt foil. The inset shows the enlarged spectra at the Pt L_2_-edge WL.

X-ray absorption spectroscopy was used to study the local electronic structure of the Pt catalysts and their interaction with the support material^38^,^39^. The normalized X-ray absorption near edge structure (XANES) spectra for both the Pt L_3_- and L_2_-edges of the ALDPt/NGNs and Pt/C catalysts are shown in Fig. 3 with comparison to a standard Pt foil. It can be seen in Fig. 3 that the threshold energy (*E*_0_) and the maximum energy (*E*_peak_) of the Pt L_3_-edge for the ALDPt/NGNs are similar to those of the corresponding metal foil, thus confirming the metallic nature of the Pt atoms and clusters on the ALDPt/NGNs samples. Furthermore, detailed examination of the spectra was conducted by qualitative and quantitative analysis of the Pt L_2_ and L_3_ white line (WL) edges. It has been shown that the area under the WL of the L_2,3_-edge of the Pt metal is directly related to the unoccupied density of states of the Pt 5*d* orbitals. This in turn has been used to correlate the catalytic activity of Pt-based electrocatalysts to changes in their local electronic structure. Close examination reveals that the intensity of the Pt L_3_ WL exhibits small differences for the ALDPt/NGNs and the Pt/C catalysts. The magnitude of the Pt WL intensity at the Pt L_3_-edge appears to increase in the order of Pt foil<Pt/C<ALD100Pt/NGNs<ALD50Pt/NGNs (Fig. 3a). In contrast, the corresponding L_2_-edge WL exhibits considerably more variation among the samples and a higher sensitivity compared with that of the Pt L_3_-edge, resulting in a different trend for the ALDPt/NGNs and the Pt/C materials. The order of increasing intensity of the WL in the Pt L_2_-edge is as follows: Pt foil<ALD100Pt/NGNs≤Pt/C<ALD50Pt/NGNs (Fig. 3b). This observation clearly confirms that for 5*d* noble metal states, the 5*d*_5/2_ and 5*d*_3/2_, demonstrate subtle but different chemical sensitivity due to the large spin–orbit coupling of the Pt 5*d* orbitals, and that both the L_3_- and L_2_-edge WLs should be used together to address the chemistry of the material^19^.

To fully understand the effect of the unoccupied densities of 5*d* states of the Pt catalysts, quantitative WL intensity analysis has been conducted on the basis of a reported method to determine the occupancy of the 5*d* states in each sample^38^,^40^,^41^ (see details in Methods). The Pt L_3_- and Pt L_2_-edge threshold and WL parameters were summarized in Table 1. The results indicate that the ALD50Pt/NGNs catalysts have the highest total unoccupied density of states of Pt 5*d* character, while the Pt/C sample has the lowest. It has been demonstrated in literature that the vacant *d*-orbitals of individual atoms play a vital role in the activity of catalysts and account for the excellent catalytic activity of single-atom catalysts^18^,^42^.

Furthermore, to study the local atomic structure of Pt using X-ray absorption spectra (XAS), the extended X-ray absorption fine structure (EXAFS) region of the X-ray absorption spectra was studied. The Fourier transforms of the EXAFS region plotted in Supplementary Fig. 10 shows several main peaks. The peak at 2.6 Å is associated with the Pt–Pt peak, which is significant in the Pt foil spectra. This peak shifts towards lower values in the Pt/C samples (2.5 Å), and is significantly dampened and shifted to higher values (2.7 Å) in the ALD-deposited samples. This is consistent with previously reported data on ALD-deposited Pt nanostructures^19^. However, as expected, due to the higher Pt content of the ALD100Pt/NGNs sample, the intensity of the Pt–Pt peak in comparison with the ALD50Pt/NGNs sample is stronger, which confirms the presence of larger clusters and the higher loading observed in the ALD100Pt/NGNs samples by ADF imaging and inductively coupled plasma-atomic emission spectroscopy results. The peaks observed around 1.7 Å in the ALD-deposited samples can be related to Pt–O or Pt–C bonds, but individual bond lengths are not distinguishable due to a similar backscattering phase. The significant intensity of Pt–O or Pt–C bonds and considerable deviation of the Pt–Pt bond of the ALD samples compared with the Pt foil indicates the presence of single Pt atoms in these samples.

## Discussion

It has been demonstrated that a strong interaction between the deposited metal and the support material plays a vital role in the stabilization of supported catalysts^43^. Despite the controlled deposition process during ALD, atomic diffusion and agglomeration are still possible and probable, if a weak interaction exists between the atoms and the support, thus resulting in the formation of large particles. This suggests the importance of the support selection when striving to prepare stable atom catalysts. It has been reported in the literature that doping the graphene lattice with N can enhance the Pt–C support interaction energy, resulting in highly stable Pt catalysts^44^,^45^,^46^. This enhanced binding energy from the incorporation of N-dopants in the graphene lattice has been attributed to creating preferred nucleation sites for metals and metal oxides^47^. In addition, it was determined in one of our previous experiments that the Pt atoms and clusters favour deposition at edge locations on NGNs, thus stabilizing the creation of Pt atoms and clusters without the formation of NPs^24^. To further examine the effects of the N-dopants on the stability of the Pt atoms and clusters observed in Fig. 1 and to expand on the understanding of the N-dopant's contribution to the HER, we have prepared ALD-deposited Pt on graphene nanosheets (GNs) for comparison. Supplementary Fig. 11 exhibits low- and high-magnification ADF images of ALD50Pt/GNs, in which there is a wide distribution of Pt sizes from atoms and clusters to larger NPs. It can be suggested that the formation of larger particles on the GNs during the ALD procedure can be attributed to the weaker interaction between the Pt and the graphene support in contrast to the N-doped graphene support. Furthermore, the HER activity was evaluated for the ALD50Pt/GNs sample using linear sweep voltammetry with a scan rate of 2 mV s^−1^ in 0.5 M H_2_SO_4_, which revealed a lower mass and specific activity in comparison with the ALD50Pt/NGNs sample (Supplementary Fig. 12). The stability of the Pt catalysts on the GNs was also confirmed using ADT tests. It was determined that the ALD50Pt/GNs sample lost 24% of its initial HER activity (Supplementary Fig. 13), while as previously reported, the ALD50Pt/NGNs only decreased its activity by 4%. Following the ADT test, the ALD50Pt/GNs sample was examined using ADF imaging, where an increased frequency of larger clusters and NPs was observed (Supplementary Figs 11 and 13). This suggests that the increased Pt particle size on the ALD50Pt/GNs sample from the ADT tests is responsible for the reduced HER activity after cycling. It can be suggested that the increased Pt particle size with cycling on the GNs is likely originating from a weaker adsorption energy, thus leading to a decreased stability of the Pt atoms, clusters and NPs, allowing them to become susceptible to degradation mechanisms^48^. Ultimately, this supports the argument that the strength of the interaction between the Pt catalysts and support increases on NGNs in comparison with GNs, which accounts for the increased stability and activity after ADT cycling.

To understand the stabilization mechanism of Pt atoms on N-doped graphene, we applied density functional theory (DFT) calculations. The DFT calculations have been carried out using a graphene (5 × 5 × 1) supercell containing 48 C atoms and 1 N atom to identify the distribution of single Pt atoms on the NGNs. All inequivalent Pt-adsorption configurations around the N atom have been considered, and the most stable configurations are shown in Supplementary Fig. 14. It can be seen that in site III, Pt is located closest to the N atom, while in site I, II, IX, X and XI the Pt atom is situated further away from the N-dopant. To determine the most stable adsorption site for the Pt atom, the adsorption energies (*E*_a_) for each site in Supplementary Fig. 14 were calculated by the following equation:


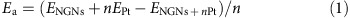


where *E*_Pt_ is the energy of an isolated Pt atom, *E*_NGNs_ is the total energy of the N-doped graphene and *E*_NGNs+*n*Pt_ is the total energy of the N-doped graphene with *n* adsorbed Pt atoms, respectively. A positive *E*_a_ indicates the successful adsorption of the Pt atoms during the N-doped graphene.

As shown in Supplementary Table 1, the Pt atom prefers to directly bond to the N-dopant, as demonstrated by the significantly larger calculated adsorption energy of 5.171 eV for a Pt atom in site III in comparison with the other adsorption sites. Moreover, the calculated Bader charge shows that 0.257 *e* is transferred from the Pt to the N atom in the N-doped graphene substrate when the system is in its most stable configuration (site III), confirming the relatively large Pt adsorption energy at this site. These results clearly suggest that the Pt prefers to adsorb to the N-dopant sites on Pt deposition.

To further examine the distribution of the Pt atoms on the N-doped graphene and their propensity to agglomerate, the energy difference between an isolated Pt atom and a Pt cluster configuration, Δ*E*_d_, was calculated, in which the total energy of the isolated configuration was used as a reference energy. In this scheme, a relatively large positive value for Δ*E*_d_ indicates that Pt atoms energetically favour the single-atom form and will tend to avoid clustering. For comparison of substrates, the production of various Pt cluster configurations on pristine graphene was also examined. As shown in Supplementary Fig. 15 and Supplementary Table 2, it is more favourable for the Pt atoms to be isolated (Δ*E*_d_=+1.145 eV) on the N-doped graphene, while the Pt atoms prefer to cluster on the pristine graphene (Δ*E*_d_=−1.357 eV). This fully supports the experimental observations of the increased size of the Pt clusters and NPs on the graphene substrate in comparison with the N-doped graphene.

A Bader charge analysis (Supplementary Table 2) has also been performed on the adsorption models for the pristine and N-doped graphene to understand the formation of bonds through charge transfer. A charge transfer (about 0.25 *e*) from the Pt atom to the support occurs on the N-doped graphene for the single Pt atom case, while almost no charge transfer exists between the Pt atom and the pristine graphene. When two Pt atoms form a dimer on the N-doped graphene, one Pt atom (Pt^1^) interacts with the graphene support, whilst the other (Pt^2^) atom sits above and bonds directly to Pt^1^. Meanwhile, Pt^1^ loses 0.521 *e* to Pt^2^, and Pt^2^ gains 0.389 *e* due to the strong bonding between Pt^1^–Pt^2^. In this cluster configuration, the surrounding N and C atoms acquire the remaining 0.132 *e*, which is less than the single-atom case. This suggests that the two Pt atoms result in an increased electron transfer to the substrate when they exist in isolation as compared with forming a dimer; therefore, the stabilization of single atoms is favoured. On the other hand, the Pt dimer adsorbed on the pristine graphene leads to an electron transfer of 0.193 *e* from Pt^1^ (0.087 *e*) and the graphene support to Pt^2^, whereas very little electron transfer occurs during the formation of the single isolated atoms on the pristine graphene. Thus, the dimer results in a system of increased stability. It can be concluded from the Bader charge and the Δ*E*_d_ that isolated atoms are the preferred form of Pt adsorption on the N-doped graphene support, while pristine graphene will favour the formation of Pt clusters.

**Figure 4:**
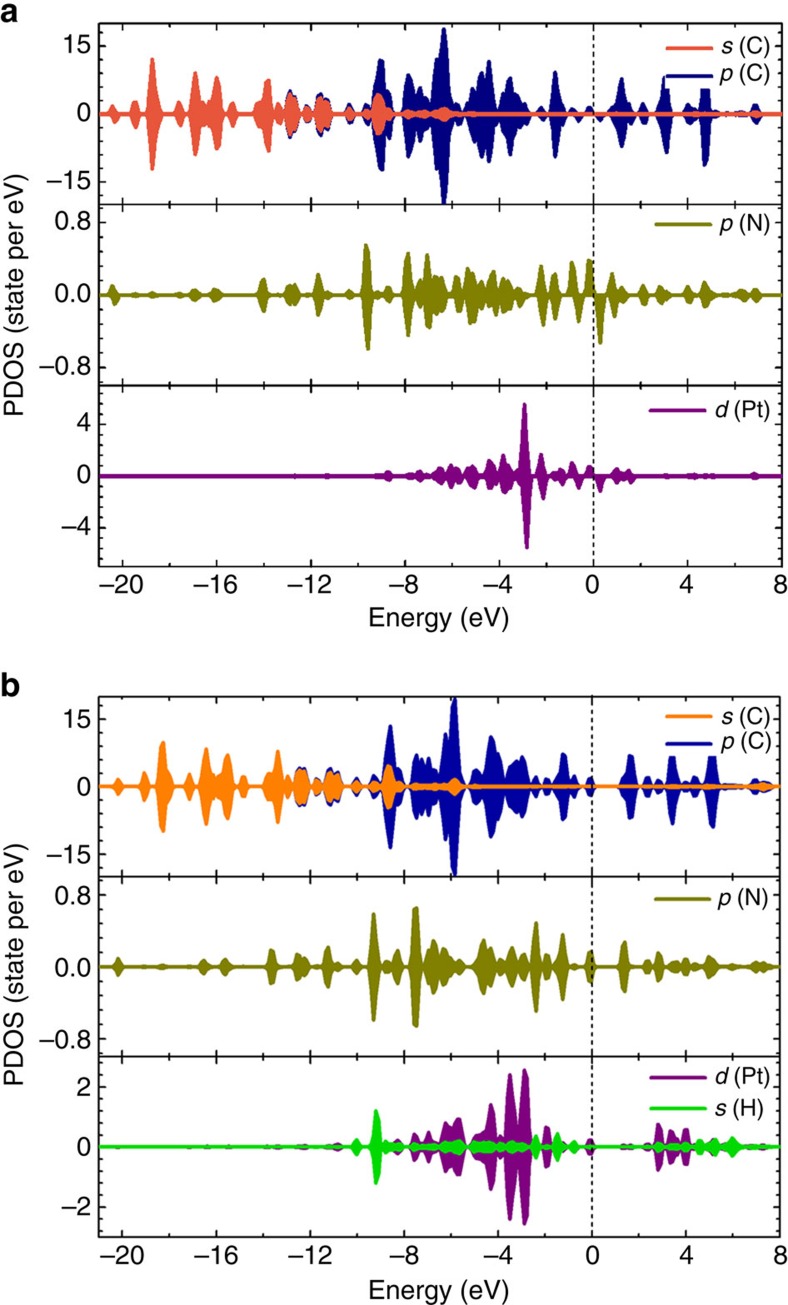
The electronic structure of a single Pt atom before and after hydrogen adsorption. Partial density of states (PDOS) of (**a**) non-H and (**b**) two H atoms adsorbed on a single Pt atom of ALDPt/NGNs. The Fermi level is shifted to zero. The upper part of the panel shows the PDOS of graphene, the middle part of the panel gives the PDOS of the N atom and the lower part of the panel exhibits the PDOS of the *d* orbital of Pt.

The chemical bonding of Pt with the N-doped graphene has also led to unique electronic properties of single Pt atoms with respect to Pt NPs, due to the charge transfer required for bond formation. The single metal atoms still carry a charge after adsorption onto the N-doped graphene substrate, which can be verified by various spectral measurements and computational modelling of the catalysts^18^,^49^,^50^,^51^. In our study, it was found that the discrete 5*d*-orbitals of the single Pt atoms are mixed with the N-2*p* orbitals around the Fermi level (Fig. 4). The calculated Bader charges (Table 2) show that the single Pt atoms are positively charged, where the N atom obtains the electron. In this case, the single Pt atoms on the N-doped graphene contain more unoccupied 5*d* densities of states. On H chemisorption (Fig. 4b), the 5*d* orbitals of the Pt atoms interact strongly with the 1*s* orbital of the H atoms, leading to electron pairing and hydride formation. In addition, more Pt (5*d*) states are found above the Fermi level, which is consistent with the calculated charge transfer from the Pt atoms to the H atoms. To fully understand the unique electronic properties of the single Pt atom catalysts for the HER, Pt clusters were also examined. As shown in Supplementary Fig. 16, the electronic properties for H adsorption on Pt clusters was investigated using a typical cluster of Pt_44_ (ref. 52). On the basis of the partial density of states and Bader charge analysis of both H adsorption on Pt_44_ and on the single Pt atoms/NGNs system (Table 2, Fig. 4 and Supplementary Fig. 16) it was found that the electron transfer from each surface Pt atom of Pt_44_ to H is <0.1 *e*, which is less than that of the single Pt atoms (+0.421 *e*). This suggests that upon H adsorption, each Pt atom of the Pt_44_ cluster remains metallic, while the single Pt atoms on the NGNs become nonmetallic through the donation of electrons to both the substrate and H atoms. This leads to the unique electronic structure of the single Pt atoms on the NGNs and is expected to be the primary reason of the increased HER activity of the ALD50Pt/NGNs sample.

Our XANES data have experimentally shown that the ALD Pt atoms do in fact have a higher total unoccupied density of 5*d* states, which agrees with the partial density of states calculation. Previous research has suggested that a change in the electronic properties of catalysts will affect the catalytic performance^52^. Thus, we carried out a series of DFT calculations to obtain a fundamental understanding of the unexpectedly high electrocatalytic activity of the single-atom catalysts. For the HER in acidic media, the overall HER pathway (H^+^+*e*^−^→1/2H_2_) can be divided into two separate pathways comprising either the Volmer–Heyrovsky or the Volmer–Tafel mechanism^53^,^54^. The first step of both pathways involves the bonding of the hydrogen ion to the catalyst, H^+^+*e*^−^→H* (Volmer reaction), where H* indicates an available surface site on the catalyst for hydrogen adsorption. In the next stage, the molecular hydrogen can be released through the Heyrovsky (H^+^+*e*^−^+H*→H_2_^↑^) or the Tafel (H*→1/2H_2_^↑^) reaction. Many previous studies^55^,^56^,^57^ have shown that the Tafel mechanism in the electrocatalytic HER is preferred at high H coverage conditions on both Pt surfaces and Pt NPs. To verify the proposed HER mechanism, we performed a computational study on the single Pt atom catalysts to gain detailed insights into the HER process. The average Pt–H distance for the H chemisorption configuration was found to be 1.623 Å, which was similar with the cases of H adsorbed on Pt surfaces and nanoclusters^58^,^59^. It was also determined that the adsorption strengths of both H and H_2_ decreased with an increase in the number of adsorbed H atoms, eventually leading to a minimum value when four H atoms were adsorbed, as shown in the Supplementary Fig. 17. In the presence of a single H atom, the most optimal orbital interaction with the Pt catalyst results in the bond formation with the most favourable Pt orbital, *d*_*z*^2^_, with a corresponding adsorption energy of 0.8 eV. As the number of interacting H atoms is increased, their bond formation with Pt is affected by the electrostatic repulsion between the H atoms. The Pt to H interaction now depends on the number of interacting H atoms, and the distinct orbitals for bond formation becomes a compromise between the H–H electrostatic repulsion and the orbital interaction of Pt–H. Specifically in the case of two H atoms, the interacting orbitals between the H atoms and the Pt catalyst become the *d*_*yz*_ and 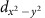 (Supplementary Fig. 18a), resulting in a corresponding adsorption energy of 0.735 eV per H. Furthermore, when three H atoms adsorb onto a single Pt atom, two of the H atoms automatically form an H_2_ dimer, due to the fact that the initial two H atoms have occupied the two empty 5*d* orbitals of the Pt catalyst. The resulting corresponding adsorption energy for three H atoms is 0.514 eV per H. Finally, as the number of H atoms reaches four (Supplementary Fig. 18c), the interaction between the Pt and H atoms are through the *d*_*yz*_ and 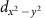 orbitals, which can effectively avoid the repulsion between the H atoms. The corresponding adsorption configuration is one in which an H_2_ dimer and two isolated H atoms are formed with an adsorption energy of 0.458 eV per H. The maximum number of adsorbed H atoms in this situation is four, as Pt no longer has empty 5*d* orbitals or physical space to allow for the interaction with an additional H atom.

We further calculated the reaction pathways and activation barriers based on the number of adsorbed H atoms to verify the proposed HER mechanism on the Pt/NGNs supports, as shown in the Supplementary Fig. 19. The calculated activation barrier was completed for the two models: a low loading of two H atoms and a high loading of four H atoms, resulting in energies of 0.664 and 0.571 eV, respectively. As previously discussed, the adsorption energy of a H atom on the single Pt atom catalyst decreases with an increase in the number of adsorbed H atoms (Supplementary Fig. 17), which promotes the formation of the H_2_ molecules. The adsorption of two H atoms on Pt (Supplementary Fig. 18a,b) results in a transformation of the hybrid orbital between the H and Pt atoms to change from 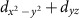 to *d*_*xy*_+*d*_*yz*_ when forming the H_2_ molecule. This orbital transformation results in the calculated energy barrier of 0.664 eV. On the other hand, when considering four H atoms, two reaction models previously outlined must be considered. The first model is the adsorption of two isolated H atoms and one H_2_ dimer, which results in the transformation of the hybrid orbital between the H and Pt atoms to change from 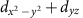 to *d*_*xy*_+*d*_*yz*_ with an energy barrier of 0.571 eV. Second, the adsorption of two H_2_ dimers on Pt results in a decreased adsorption energy of 0.336 eV per H in comparison with the four H atom model, which should further promote the ease of H_2_ production on the Pt atom. Importantly, it should be noted that the energy barrier for H_2_ formation is lower when four H atoms are present, thus clarifying that the H_2_ formation on single Pt atom catalysts is preferred at high H coverage. Moreover, the calculated activation barriers for both the two H and four H atoms models is smaller than that of the conventional Pt (111) surface (∼0.85 eV)^56^,^57^. The decreased activation barrier calculated for the HER of the single Pt atoms on N-doped graphene is consistent with our experimentally observed fast HER kinetics.

For practical applications concerning single-atom catalysts, a high activity and good stability are paramount to ensure a competitive performance is achieved in comparison with conventional NP catalysts. Furthermore, the performance will rely on achieving a high density of single atoms to ensure that the number of active sites is not reduced. To examine this effect, the performance of 50 ALD cycles was compared with 25 cycles. It was found that the current density of the ALD25Pt/NGNs sample was below that of commercial Pt/C catalysts, resulting in the lowest current density shown in Supplementary Fig. 20. The low specific activity of the ALD25Pt/NGNs sample may result from a low density of single Pt atoms/clusters (Pt loading of 0.19%) on the N-doped graphene. The low Pt density could be an effect of the low cycle number and nucleation delay^60^ in the first few ALD cycles.

In conclusion, we fabricated novel Pt catalysts supported by NGNs for the HER using the ALD technique. The size of the Pt catalysts ranged from single atoms, sub-nanometre clusters, to NPs, which were precisely controlled by adjusting the number of ALD cycles. Single Pt atoms and clusters showed exceptionally high activity and stability as HER catalysts compared with commercial Pt/C catalysts. The remarkable performance of the single Pt atoms and clusters arise from their small size and the unique electronic structure originating from of the adsorption of the single Pt atoms on the N-doped graphene, as confirmed by XANES and DFT analysis. Our work provides a promising approach for the design of highly active and stable next-generation catalysts based on single Pt atoms and clusters, which have a great potential to reduce the high cost of industrial commercial noble-metal catalysts.

## Methods

### Synthesis of GNs and NGNs

Graphite oxide was first obtained by a modified Hummers method previously reported by our group^61^,^62^. The received graphite oxide was then rapidly exfoliated via a thermal treatment at 1,050 °C under Ar atmosphere, yielding the product of GNs. NGNs were prepared by post-heating the graphene under high purity ammonia mixed with Ar at 900 °C for 10 min.

### ALD synthesis of Pt on NGNs

Pt was deposited on the NGNs by ALD (Savannah 100, Cambridge Nanotechnology Inc., USA) using MeCpPtMe_3_ and O_2_ as precursors and a procedure similar to one previously reported^19^. High-purity N_2_ (99.9995%) was used as both a purging gas and carrier gas. The powder NGNs or GNs were placed in a container inside the ALD reactor chamber. The deposition temperature was 250 °C, while the container for MeCpPtMe_3_ was kept at 65 °C to provide a steady-state flux of MeCpPtMe_3_ to the reactor. Gas lines were held at 100 °C to avoid precursor condensation. For each ALD cycle, 1 s of the MeCpPtMe_3_ pulse and 5 s of the O_2_ pulse were separated by a 20 s N_2_ purge. The size, density and distribution of the Pt catalysts on the NGNs or GNs were precisely controlled by adjusting the number of ALD cycles.

### Physical characterization

The ADF STEM images were acquired using an FEI Titan 80–300 Cubed TEM equipped with a monochromator, hexapole-based aberration corrector (Corrected Electron Optical Systems GmbH) in both the probe and imaging lenses, and a high-brightness gun (XFEG) operated with an 80 kV accelerating voltage. High-resolution transmission electron microscopy was performed using an FEI 80–300 Cryo-Twin TEM with a Schottky field emission gun under negative C_s_ imaging conditions using a hexapole-based aberration corrector in the imaging lens and the electron source operated at 300 kV accelerating voltage. Samples were baked under vacuum at 100 °C before imaging to prevent beam contamination.

### X-ray absorption spectroscopy

XANES measurements of the Pt L_2_-edge and the Pt L_3_-edge were conducted on the 06ID superconducting wiggler sourced hard X-ray microanalysis beamline at the Canadian Light Source. Each sample spectra were collected in fluorescence yield using a solid-state detector, while the high-purity Pt metal foil spectra were collected in transmission mode for comparison and monochromatic energy calibration.

WL intensity analysis was conducted based on previous research^38^,^40^. In this method, the Pt L_3_-edge WL intensity was obtained by subtracting the Pt L_3_-edge XANES from the corresponding XANES of Au. The area under the difference curve was integrated between the two vertical bars, and Δ*A*_3_ and Δ*A*_2_ were calculated using the following expressions:


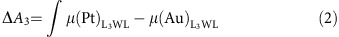



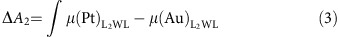


According to Sham *et al*.^40^, these values are related to the following theoretical expressions:


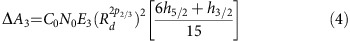



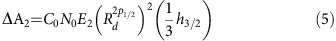


where *C*_0_=4*πr*^2^*α*/3 (*α* is the fine structure constant), *N*_0_ is the density of Pt atoms, *h*_*j*_ is the 5*d* hole counts, *R* is the radial transition matrix element, and *E*_2_ and *E*_3_ are the corresponding edge thresholds (*E*_o_) for the L_2_- and L_3_-edges, respectively. By assuming that the *R* terms are similar for both edges


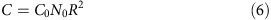


and with this approximation


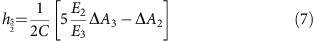



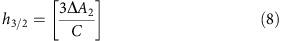


The *C* value for these equations was previously derived for the Pt metal as 7.484 × 10^4^ cm^−1^ (ref. 63).

### Electrochemical characterization

A three-compartment cell was used for the electrochemical measurements with a glassy carbon rotating-disk electrode (Pine Instruments) as the working electrode. Hg/Hg_2_SO_4_ electrode and a Pt wire were used as the reference and the counter electrode, respectively. The potentials presented in this study are referred with respect to RHE.

The catalyst dispersions were prepared by mixing 3 mg of catalyst in a 2 ml aqueous solution containing 1 ml of isopropyl alcohol and 30 μl of 5 wt% Nafion solution. Following the solution preparation, the mixture was ultrasonicated for 30 min. Next, the working electrode was created by transferring 10 μl of the aqueous catalyst dispersion onto the glassy carbon rotating disk electrode (0.196 cm^2^). The working electrode was rotated at 1,600 r.p.m. to remove the H_2_ gas bubbles formed at the catalyst surface.

### Computational methods

Our calculations are performed based on DFT calculations, as implemented in the Vienna ab initio package^64^,^65^. The general gradient approximation of Perdew–Burke–Ernzerhof is adopted for the exchange-correlation functional^66^. Moreover, the electron wave functions were expanded by a plane wave cutoff of 400 eV. The (5 × 5 × 1) supercell N-doped graphene contains 48 C atoms and 1 N atom was constructed by a periodic boundary condition, and the vacuum layers were set to be larger than 20 Å to avoid periodic interaction. Reciprocal space was performed by the Monkhorst–Pack special *k*-point scheme with 5 × 5 × 1 grid meshes for structure relaxation for the Pt adsorbed on N-doped graphene. Atomic relaxation was performed until the total energy variation was smaller than 10^−6^ eV and all forces on each atom were <0.01 eV Å^−1^. The van der Waals density functional^67^,^68^ approach was used to evaluate the effect of the van der Waals interaction^68^.

We determined the free energy barrier for the HER on the Pt adsorbed on N-doped graphene according to the Volmer–Tafel route using the climb image nudged elastic band method^69^. A set of images (*N*=9) is uniformly distributed along the reaction path connecting the initial and final states optimized in our simulation. To ensure the continuity of the reaction path, the images are coupled with elastic forces, and each intermediate state was fully relaxed in the hyperspace perpendicular to the reaction coordinate. The Bader charge analysis^70^ was performed to quantitatively estimate the amount of charge transfer between the adsorbed Pt (or H) and the N-doped graphene.

### Date availability

The data that support the findings of this study are available from the corresponding authors on request.

## Additional information

**How to cite this article**: Cheng, N. *et al*. Platinum single-atom and cluster catalysis of the hydrogen evolution reaction. *Nat. Commun.* **7**, 13638 doi: 10.1038/ncomms13638 (2016).

**Publisher's note**: Springer Nature remains neutral with regard to jurisdictional claims in published maps and institutional affiliations.

## Supplementary Material

Supplementary Information Supplementary Figures 1-20 and Supplementary Tables 1-2.

## Figures and Tables

**Table 1:**
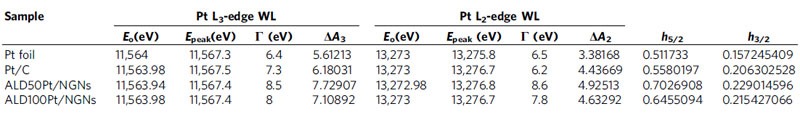
Pt L_3_-edge and Pt L_2_-edge WL parameters.

**Table 2:** Calculated bond lengths and the Bader charge of the non-H and two H atoms adsorbed on the single Pt atom of ALDPt/NGNs.

	***l***_**Pt–N**_ **(Å)**	***l***_**Pt–H**_ **(Å)**	**Bader charge (*****e*****)**		
			**Pt**	**N**	**H**
Hydrogen adsorption	2.105	1.6591.580	+0.421	−1.202	−0.073−0.193
Non-H	2.309	—	+0.278	−1.076	—

Here the bond lengths of Pt–N (*l*_Pt–N_) and Pt–H (*l*_Pt–H_) are listed. The Bader charge for both Pt, N and H are shown before and after H adsorption.
