# Supplementary material for: Platinum single-atom and cluster catalysis of the hydrogen evolution reaction
Source: Nat Commun. 2016 Nov 30;7:13638. doi: 10.1038/ncomms13638 (PMC5141386; doi:10.1038/ncomms13638)
Supplement: Supplementary Information — Supplementary Figures 1-20 and Supplementary Tables 1-2. [file ncomms13638-s1.pdf]

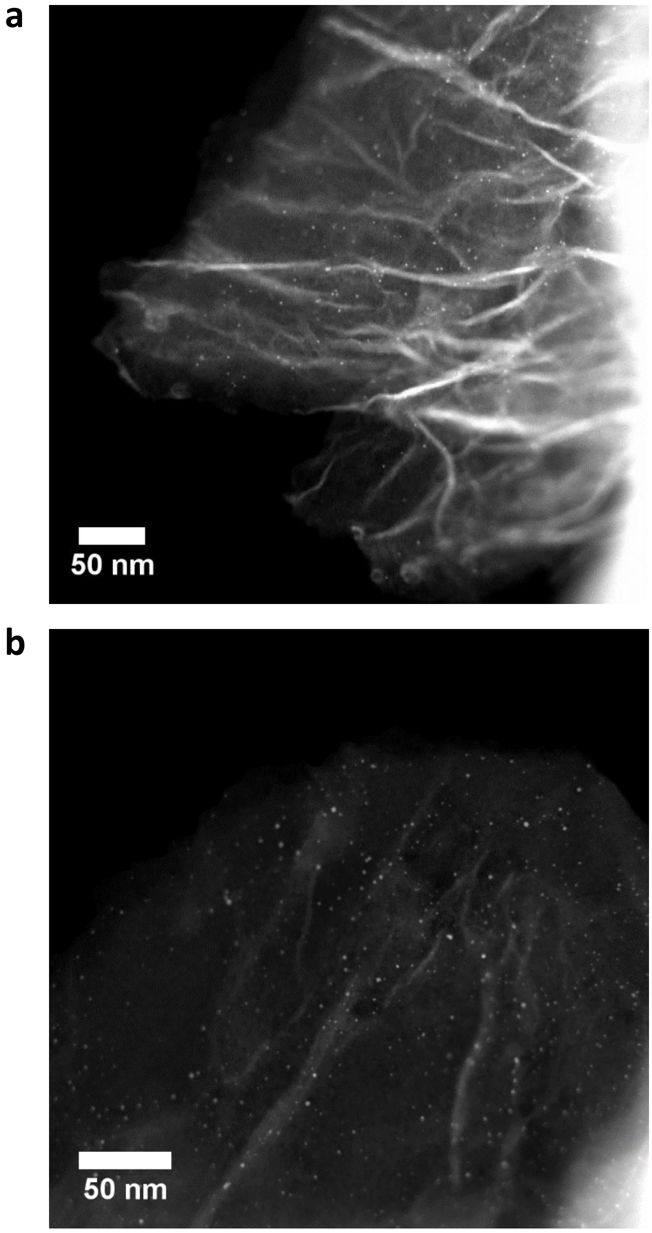

Supplementary Figure 1 | ADF STEM images of ALDPt/NGNs. The ADF images were acquired from NGNs with (**a**) 50 Pt ALD cycles and (**b**) 100 Pt ALD cycles, respectively.

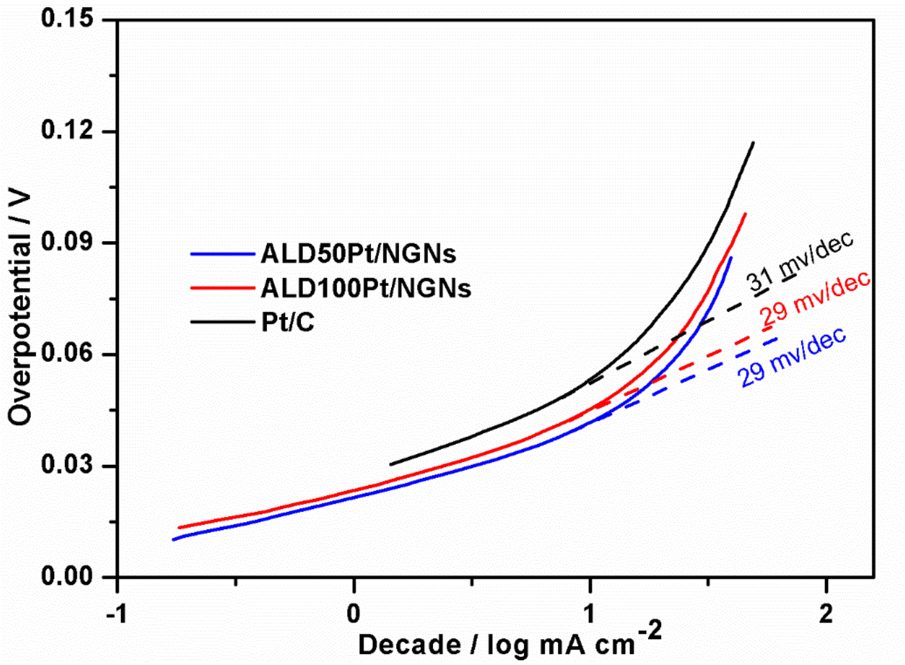

Supplementary Figure 2 | Electrocatalytic experiments on ALDPt/NGNs. The Tafel plots for the ALDPt/NGNs and the Pt/C catalysts.

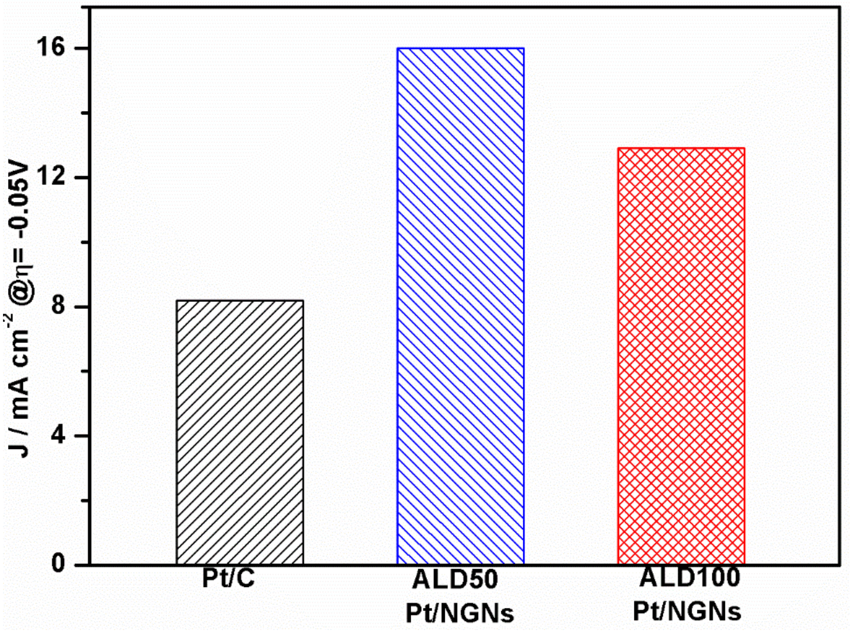

Supplementary Figure 3 | Specific activity measurements. Specific activity at 0.05 V (vs. RHE) of the ALDPt/NGNs and Pt/C catalysts for the HER.

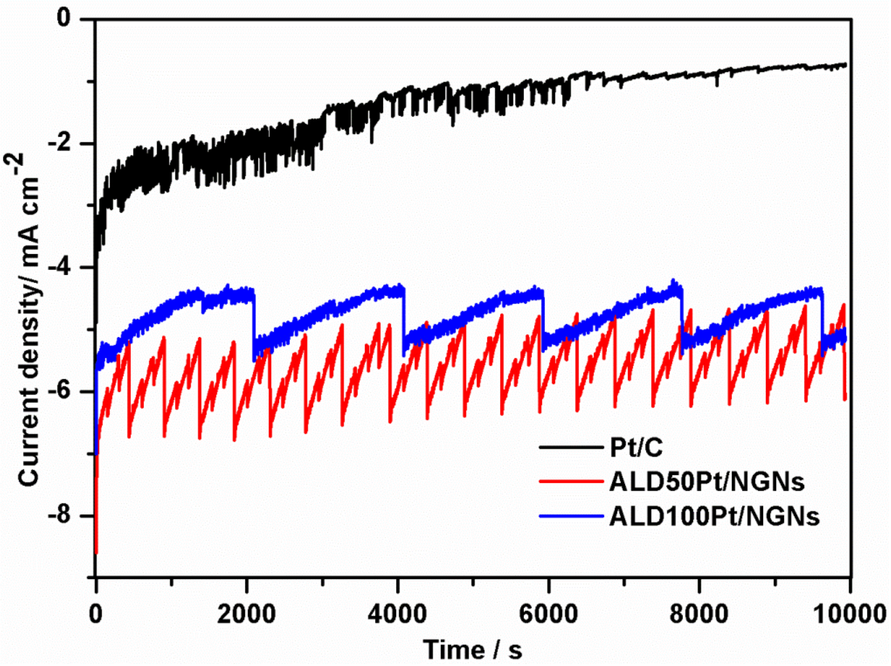

Supplementary Figure 4 | Stability measurements. Stability of the ALDPt/NGNs and Pt/C catalysts for the HER at 0.04 V (vs. RHE) for 9950s.

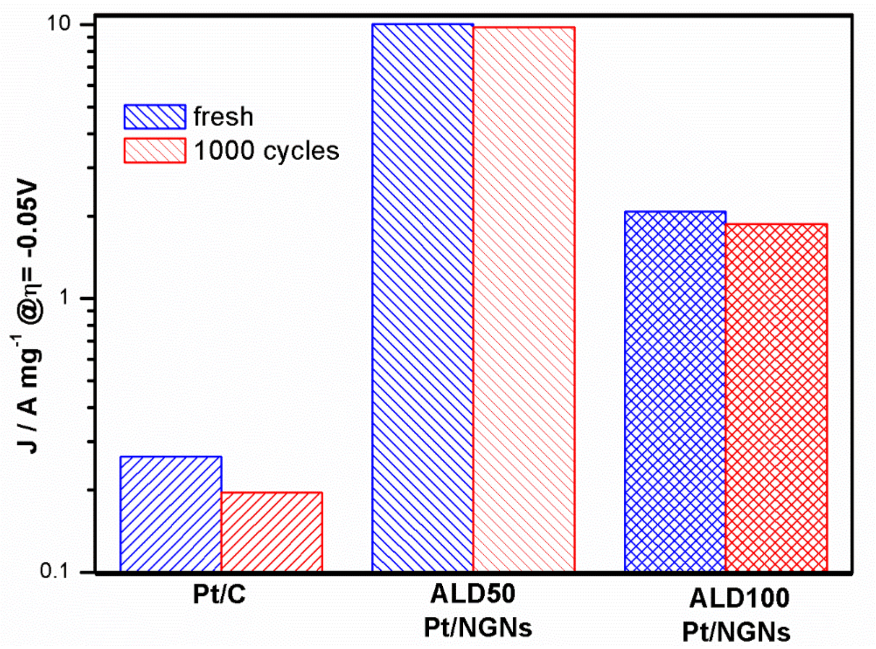

Supplementary Figure 5 | Mass activity measurements. Mass activity at 0.05 V (vs. RHE) of the ALDPt/NGNs and Pt/C catalysts for the HER before and after ADT.

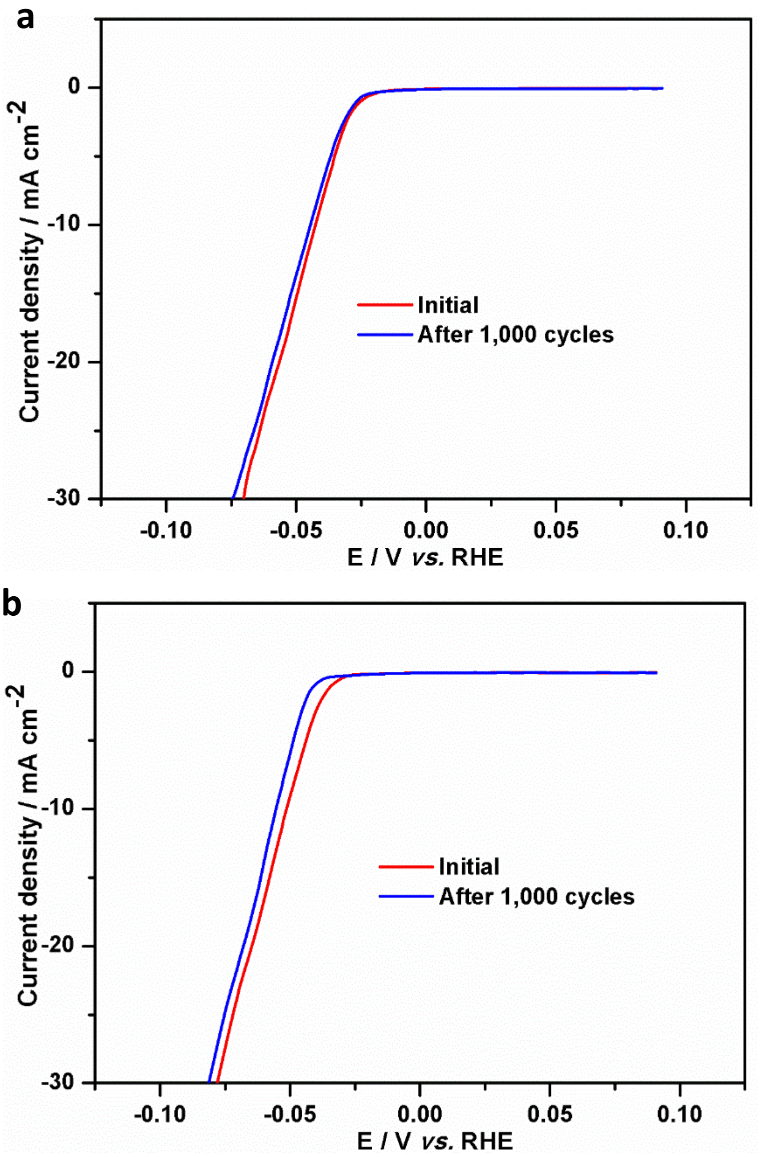

Supplementary Figure 6 | Durability measurements. (**a**) ALD100Pt/NGNs and (**b**) Pt/C catalysts. The polarization curves were recorded for the first cycle and after 1000 CV sweeps between +0.4 and -0.15 V (vs. RHE) at 100 mV s^{-1}. All the polarization curves were performed in 0.5 M H_2SO_4 at a scan rate of 2 mV s^{-1}.

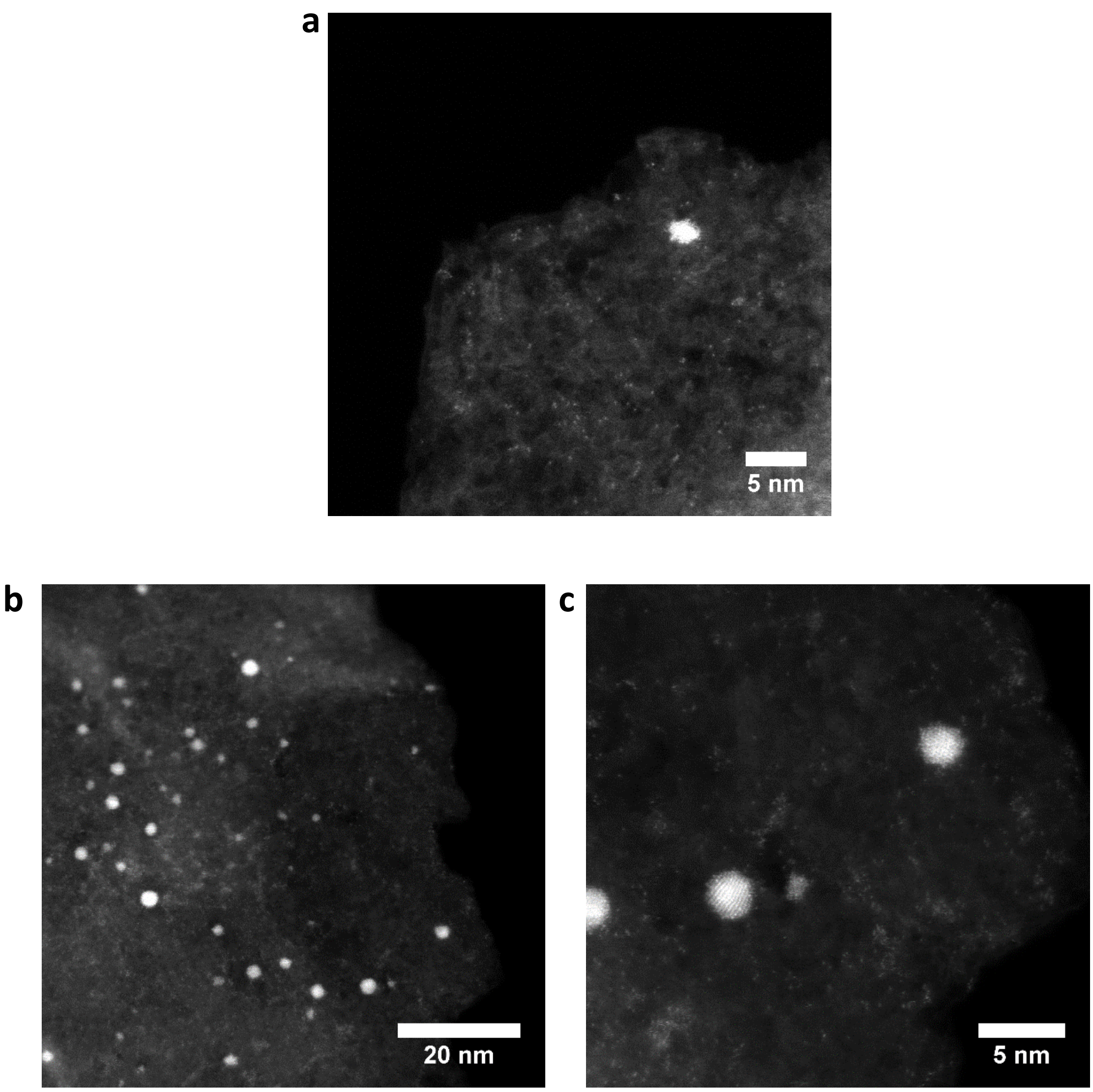

Supplementary Figure 7 | ADF STEM images after stability measurements of ALDPt/NGNs. The ADF images of NGNs with (**a**) 50 and (**b** and **c**) 100 Pt ALD cycles, respectively were acquired after ADT.

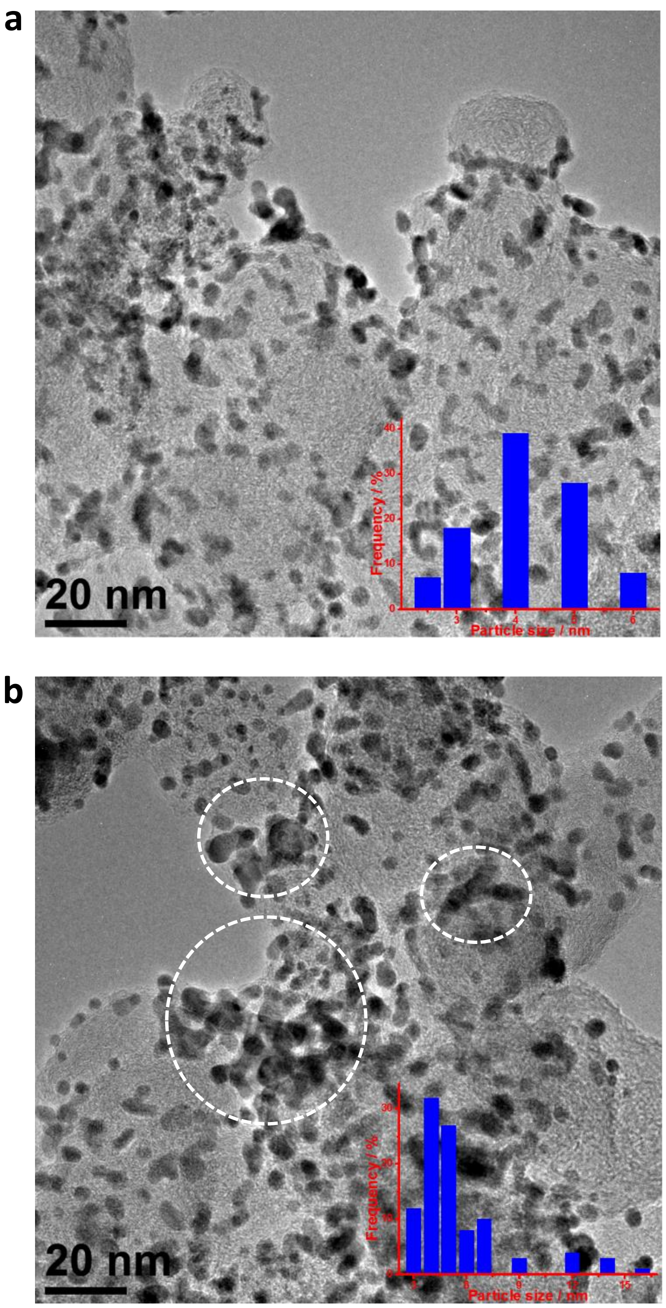

Supplementary Figure 8 | HRTEM images of conventional catalysts. Images of Pt/C catalysts (**a**) before and (**b**) after ADT.

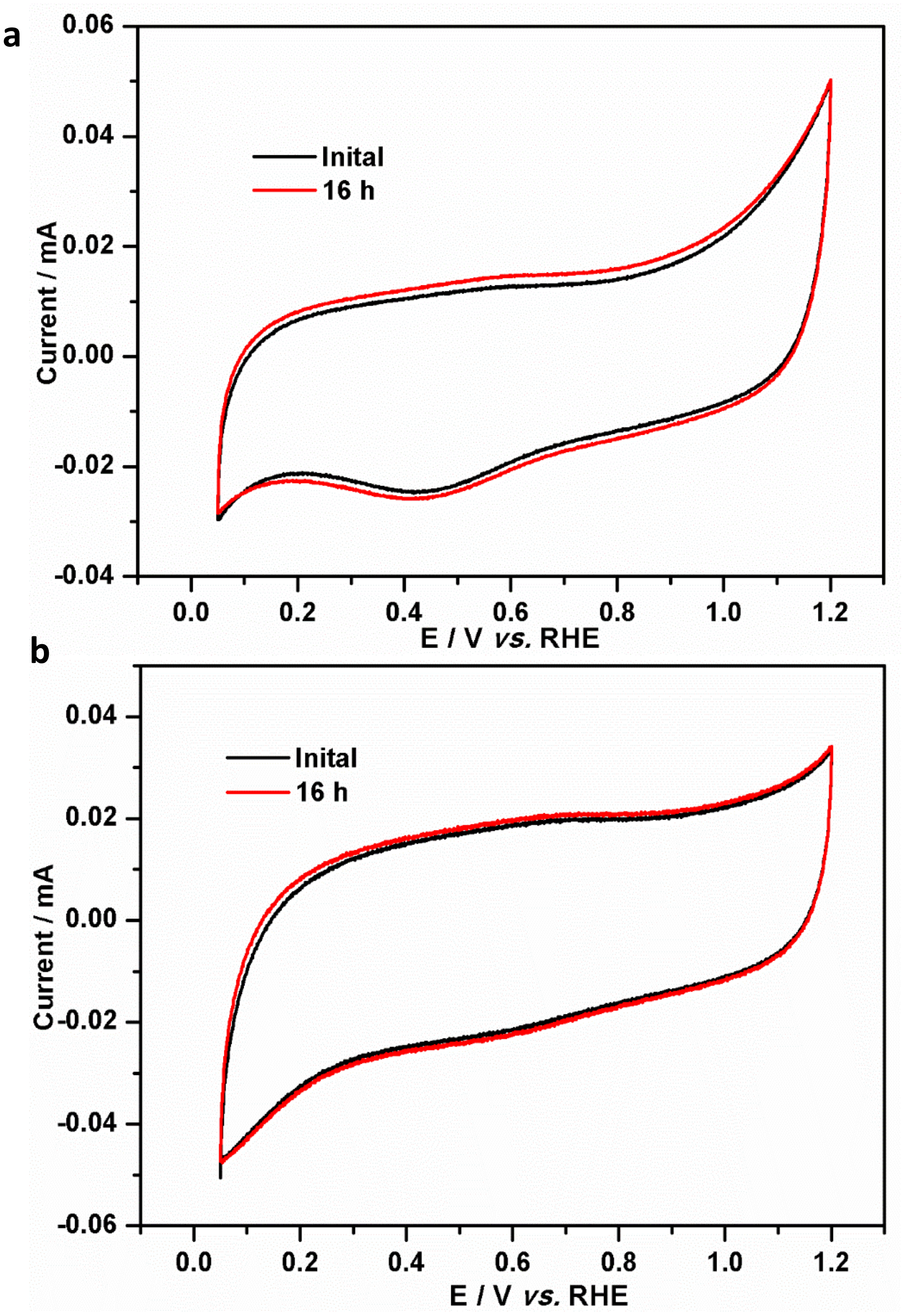

Supplementary Figure 9 | CV curves. The CV curves for (**a**) carbon black (Vulcan XC-72) and (**b**) NGNs when held at 0.4 V for 16 h.

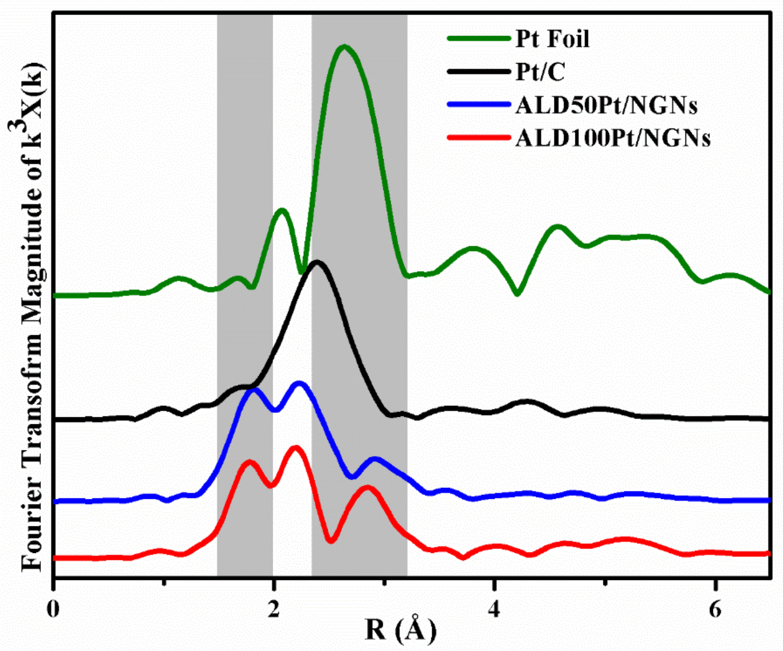

Supplementary Figure 10| K3 weighted Fourier transform spectra. Spectra acquired from EXAFS of ALDPt/NGNs catalysts, Pt/C catalysts, and a Pt foil.

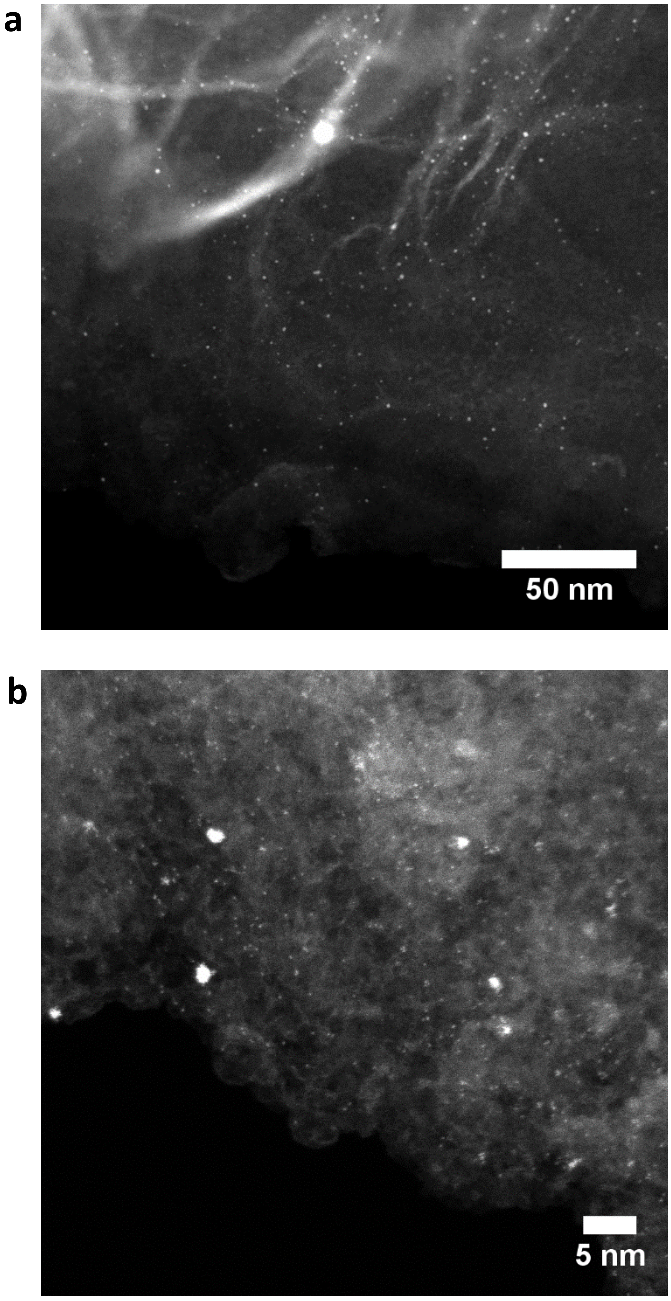

Supplementary Figure 11 | ADF STEM images of GNs. ALD50Pt/GNs samples at low (**a**) and high (**b**) magnification.

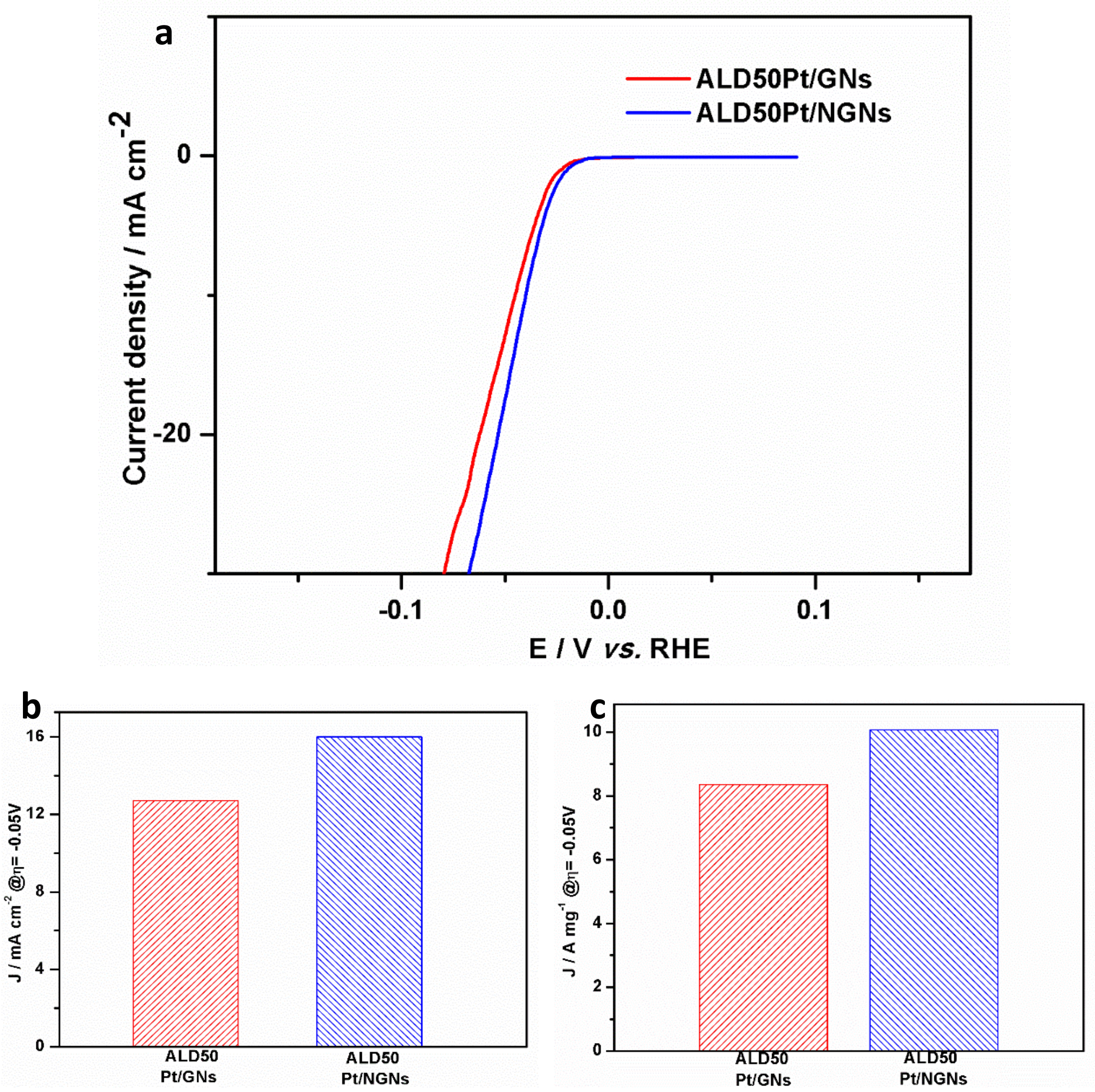

Supplementary Figure 12 | HER polarization curve measurements. The HER (**a**) polarization curves for ALD50Pt/GNs and ALD50Pt/NGNs catalysts were acquired by LSV with a scan rate of 2 mV s^{-1} in 0.5 M H_2SO_4 at room temperature. N_2 was purged before the measurements. The (**b**) Specific activity and the (**c**) mass activity were measured at 0.05 V (vs. RHE) for the ALD50Pt/GNs and ALD50Pt/NGNs catalysts for the HER.

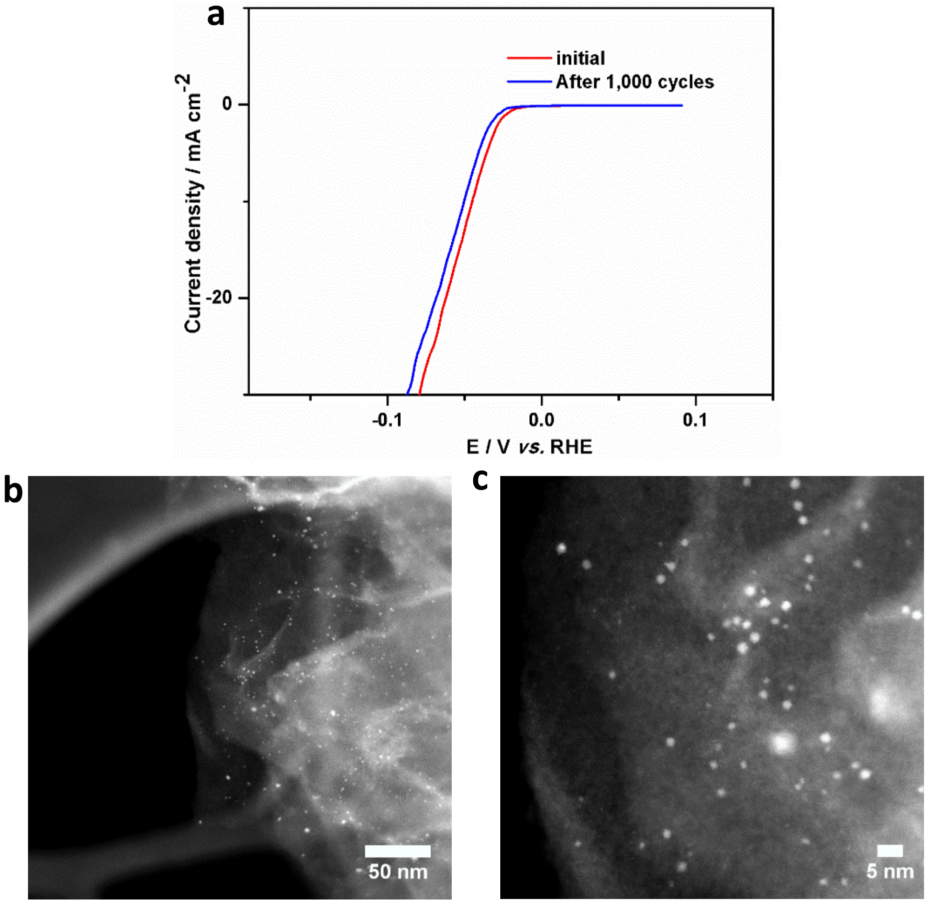

Supplementary Figure 13 | Durability measurements of ALD50Pt/GNs. (**a**) The polarization curves were recorded for the first cycle and after 1000 CV sweeps between +0.4 and -0.15 V (vs. RHE) at 100 mV s^{-1}. The polarization curves were performed in 0.5 M H_2SO_4 at a scan rate of 2 mV s^{-1}. ADF images were acquired at a low (**b**) and high (**c**) magnification after the ALD50Pt/GNs were cycled.

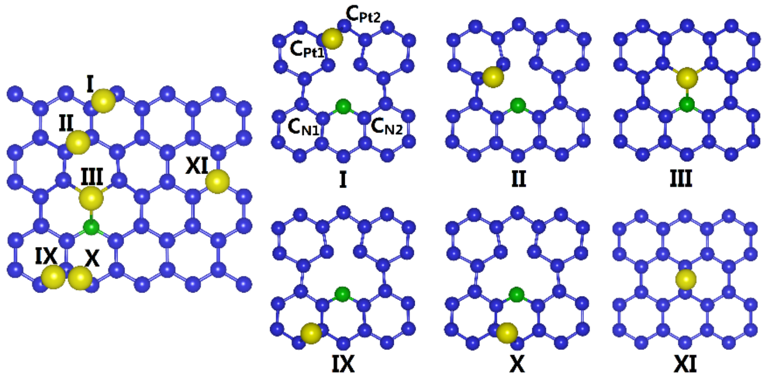

Supplementary Figure 14| Optimized stable structures of Pt atoms adsorbed on different sites on the N-doped graphene. The left most image shows the combined optimized structures the Pt atoms on the N-doped graphene, while the individual structures are labeled as I, II, III, IX, X, and XI on the right. Blue, green and yellow indicate C, N and Pt atoms, respectively.

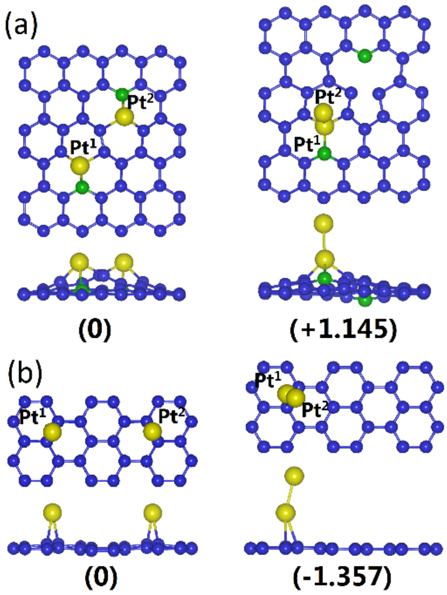

Supplementary Figure 15 |Illustration of Pt configurations on N-doped and pristine graphene. Illustration of a Pt cluster (right) and isolated (left) configuration on (**a**) N-doped graphene and (**b**) pristine graphene, respectively. The energy of the isolated configuration was taken as a zero reference for comparison. Blue, green and yellow indicate C, N and Pt atoms, respectively.

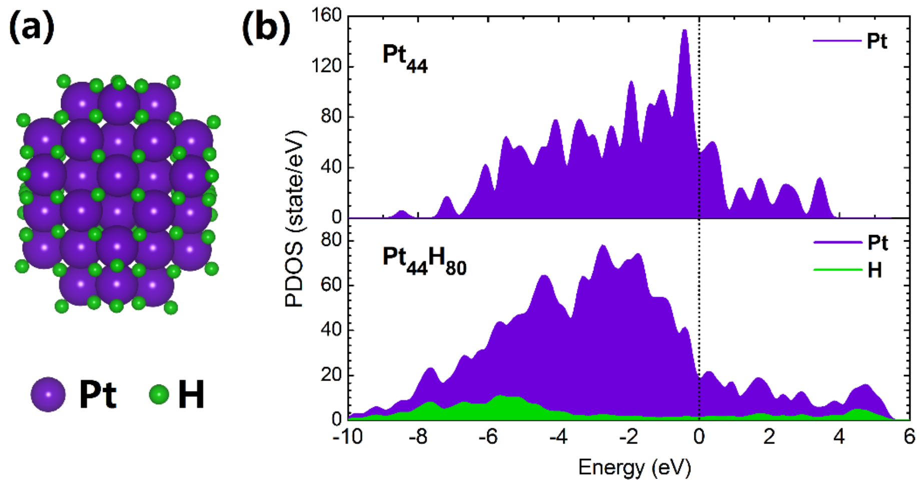

Supplementary Figure 16 | The interaction between H and a Pt cluster. (**a**) Optimized structure of $Pt_{44}H_{80}$, and (**b**) is the PDOS of a pure (top panel) and H chemisorbed (bottom panel) Pt_{44} catalysts. The Fermi level is shifted to zero.

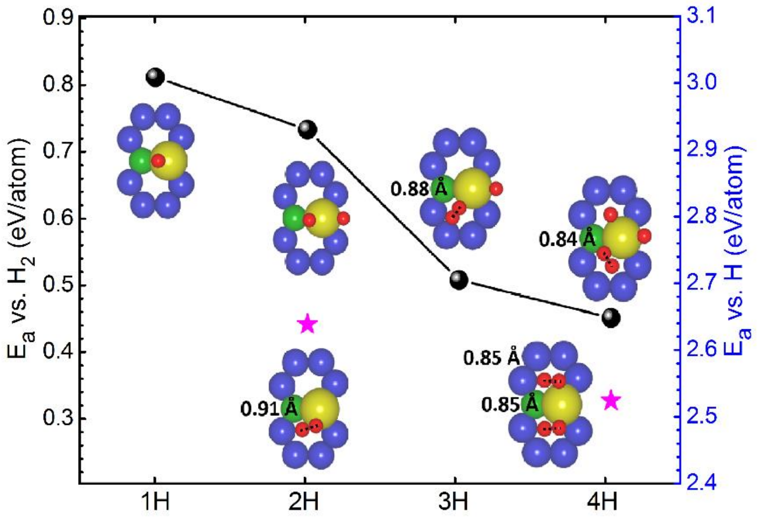

Supplementary Figure 17 | Calculated adsorption energies of H atoms as a function of the H coverages (from one H atom to four H atoms) on the single Pt atom catalysts with a NGNs support. Blue, green, yellow and red indicate C, N, Pt and H atoms, respectively. The H-H distances are also shown in the figure. The black spheres represent the most stable adsorption structure under different H concentrations. The magenta star indicates the two and four H atom adsorption configurations which forms one and two H_2 dimers on a single Pt atom, respectively. Here the distance between the H atoms is also labeled for the H_2 dimer on the single Pt atom. The adsorption energies (E_a) related to both H_2 and H were calculated by:

$$E_a = [E_{NGNs+Pt} + \frac{n}{2} E_{H_2} (or\ nE_H) - E_{NGNs+Pt+nH}]/n. \quad \text{(Supplementary 1)}$$

The left scale shows the calculated adsorption energy relative to the H_2 molecule, and the right scale gives the corresponding adsorption energy relative to the isolated H atom for comparison. In the main context, we discuss the H adsorption energies relative to the H_2 molecule.

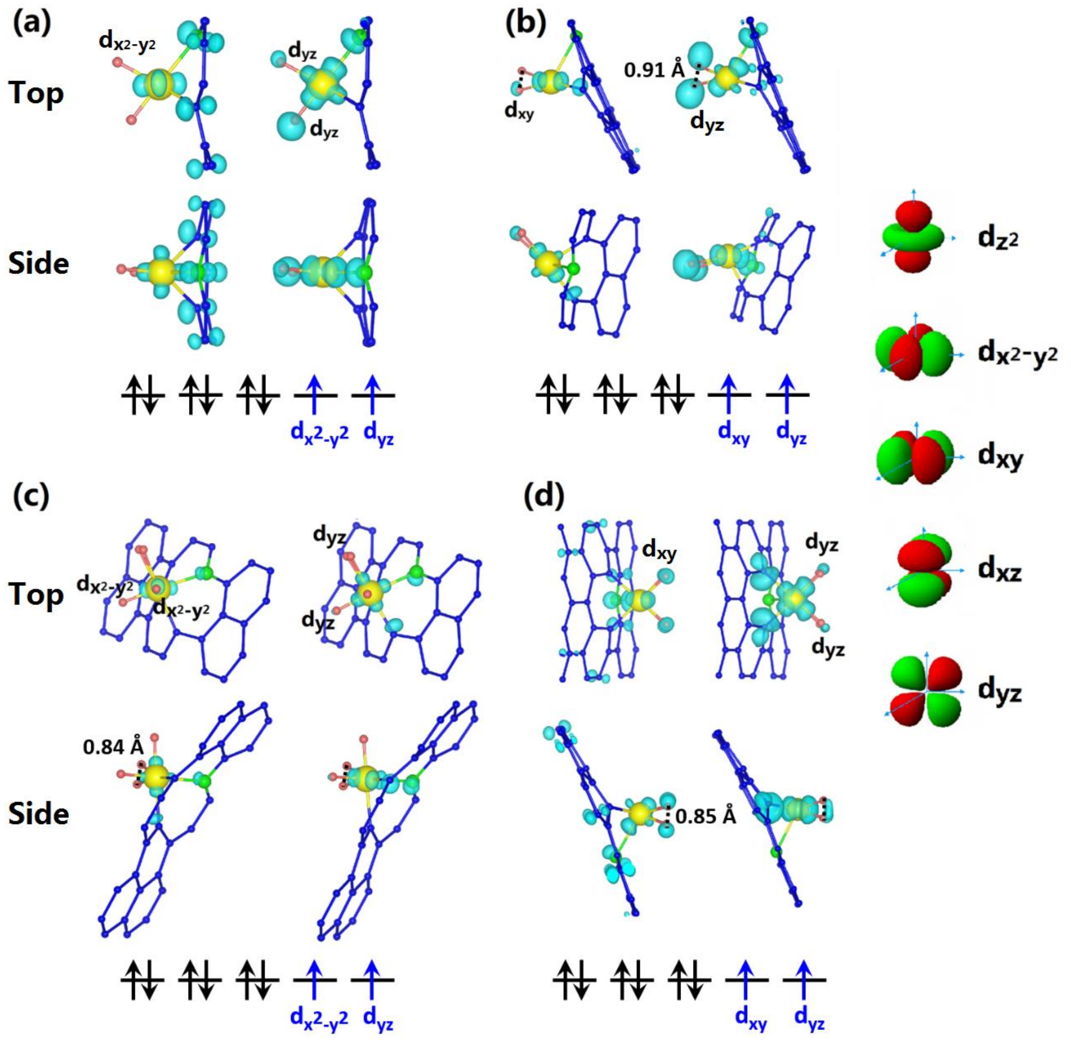

Supplementary Figure 18 | The hybrid orbitals between the Pt and H atoms for the two H and four H atom cases. (**a**) and (**b**) have two H atoms adsorbed on the single Pt atom catalyst: (**a**) two isolated H atoms, H + H, (**b**) one H_2 dimer. (**c**) and (**d**) have fours H atoms: (**c**) one H_2 dimer plus two isolated H atoms, (**d**) two H_2 dimers. The left panel shows the first interacting orbital, and the right panel gives the second interacting orbital. The schematics for the orbital filling are shown under each partial charge density, and the typical characteristic of five *d* orbitals (d_{xy}, d_{yz}, d_{xz}, $d_{x^2-y^2}$ and d_{z^2},) are also shown on the right side as well. The different orientations are used to show the orbitals clearly. The C, N, Pt and H atoms are represented by blue, green, yellow and red, respectively, and the H-H distances are also shown. The isovalues of the charge densities are defined as 0.03 e $Å^{-3}$ in all cases.

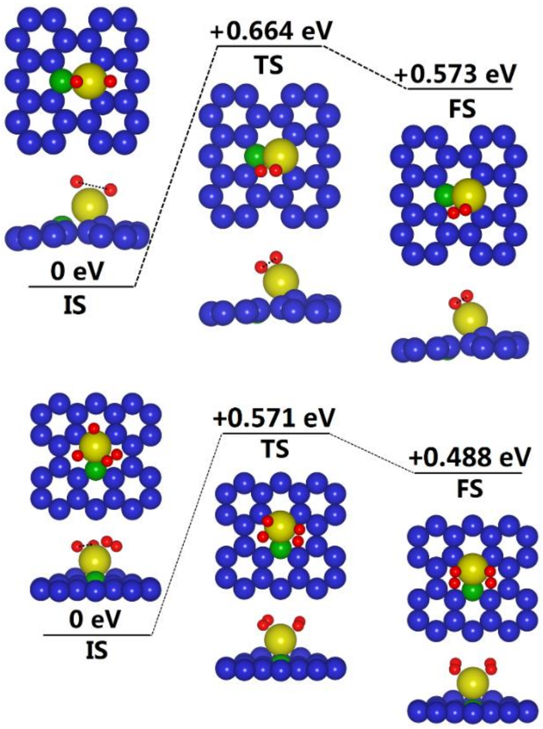

Supplementary Figure 19 | HER mechanism. The calculated reaction barriers and the optimized atomic structures for the HER are shown with two H (upper panel) and four H atoms (lower panel) on the Pt/NGNs. Here, IS, TS, and FS represents the initial state, transition state, and final state, respectively. Blue, green, yellow and red indicate C, N, Pt and H atoms, respectively.

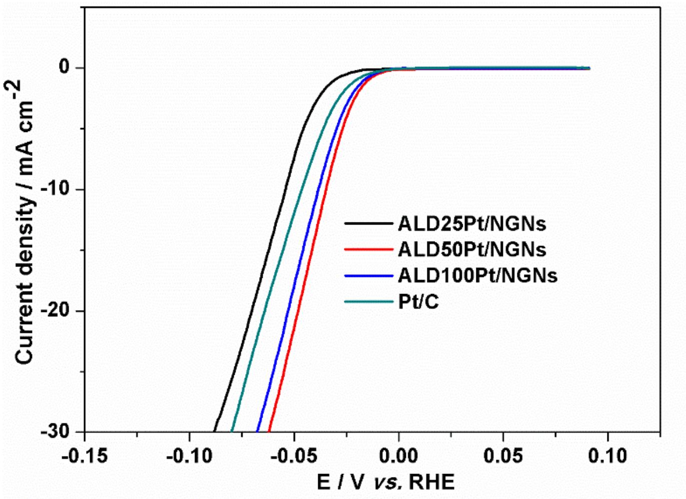

Supplementary Figure 20 |HER polarization curves compared for varying number of ALD Pt cycles. HER polarization curves were compared for varying number of ALD Pt cycles on N-graphene to Pt/C catalysts. The polarization curves were acquired by LSV with a scan rate of 2 mV s^{-1} in 0.5 M H_2SO_4 at room temperature.

Supplementary Table 1 | Calculated Pt adsorption energy (E_a), Bader charge, and the Pt-N distance of different Pt-adsorption sites on the N-doped graphene. The corresponding structures can be found in Supplementary Fig. 14. A positive Bader charge indicates the loss of electrons, where a negative value denotes that electrons were gained.

	E_a (eV)	Bader charge (e)				Pt-N
		Pt	N	C_{Pt}	C_N	distance (Å)
I	1.971	+0.01	-1.207	-0.026/-0.057	+0.593/+0.584	4.872
II	2.811	-0.003	-1.203	+0.050/-0.006	+0.595/+0.638	3.343
**III**	**5.171**	**+0.257**	**-1.137**	**-0.015/-0.069**	**+0.453/+0.617**	**2.310**
IX	1.823	-0.012	-1.208	+0.063/-0.044	+0.570/+0.565	3.920
X	1.788	+0.007	-1.238	+0.043/-0.131	+0.509/+0.599	3.504
XI	1.769	+0.005	-	+0.115/-0.062	-	-

Supplementary Table 2 | Calculated difference energy (ΔE_d), average Pt-Pt bond length ($l_{Pt\text{-}Pt}$), Pt-C bond length ($l_{Pt\text{-}C}$), Pt-N bond length ($l_{Pt\text{-}N}$) and the Bader charge of Pt in an isolated and clustered configuration. The corresponding structures can be found in Supplementary Fig. 15. A positive Bader charge indicates the loss of electrons, where the negative value denotes that electrons were gained.

		E_d (eV)	$l_{Pt\text{-}Pt}$ (Å)	$l_{Pt\text{-}C}$ (Å)	$l_{Pt\text{-}N}$ (Å)	Bader charge (e)		
						Pt_1	Pt_2	N
Isolated configuration	N-doped	0	4.181	1.937	2.069	+0.253	+0.281	-1.161/-1.152
	pristine	0	6.405	2.099	-	-0.007	-0.002	-
Cluster configuration	N-doped	+1.145	2.323	1.945	2.239	+0.521	-0.389	-1.225/-0.153
	pristine	-1.357	2.358	2.259	-	+0.087	-0.193	-
